# The ^68^Ga-siderophore approach to infection imaging: evaluation of [^68^Ga]Ga-DFO in patients with vascular graft infection

**DOI:** 10.1007/s00259-026-07831-4

**Published:** 2026-03-06

**Authors:** Sally F Barrington, Margaret Cooper, Georgios Krokos, Zilin Yu, Afnan MF Darwesh, Victoria Gibson, Ronan Tegala, Julia E Blower, Maria Dibua, Armidita Jacob, Joemon John, Sofia Pereira, Laura Cole, Nicholas Price, Morad Sallam, Philip J Blower

**Affiliations:** 1 King’s College London and Guy’s and St Thomas’ PET Centre, School of Biomedical Engineering and Imaging Sciences, King’s College London, King’s Health Partners, SE1 7EH, London, UK; 2 Department of Radiologic Sciences, Faculty of Applied Medical Sciences, King Abdulaziz University, Jeddah, Saudi Arabia; 3 Department of Nuclear Medicine, Guy’s & St Thomas’ NHS Foundation Trust, London, UK; 4 Department of Vascular Surgery Guy’s &, St Thomas’ NHS Foundation Trust, London, UK; 5 Directorate of Infection, Guy’s & St Thomas’ NHS Foundation Trust, London, UK

**Keywords:** bacterial infection imaging, gallium radioisotopes, positron emission tomography computed tomography, prosthetic vascular graft infection, radiopharmaceuticals

## Abstract

**Purpose:**

Gallium-68 complexes with siderophores, typically isostructural with their iron(III) analogues and recognized by microbial Fe(III)-siderophore complex receptors, are candidates for PET imaging of microbial infection. Here we evaluate [^68^Ga]Ga-desferrioxamine-B complex ([^68^Ga]Ga-DFO) for imaging in patients with infected aortic grafts.

**Methods:**

The trial was registered on clinical trials.gov, NCT05285072 registered 16 March 2022. [^68^Ga]Ga-DFO was produced from ^68^Ga-generator eluate in acetate buffer and characterized by radioHPLC, iTLC and LCMS. Its stability in human serum was evaluated in vitro by protein precipitation and size exclusion chromatography, and its biodistribution, pharmacokinetics and dosimetry determined by PET imaging in healthy mice. PET imaging in two patients with aortic graft infections was performed over 90 min, with blood and urine sampling in one patient.

**Results:**

Analysis of [^68^Ga]Ga-DFO identified it as a 1:1 complex of ^68^Ga with DFO, with > 95% radiochemical purity, stable in human serum in the absence of bicarbonate at high DFO: protein ratio, but not in the presence of bicarbonate with low DFO: protein ratio. It was rapidly cleared renally from healthy mice but in humans, clearance was much slower consistent with significant protein binding. There was no specific uptake at infection sites identified by [^18^F]FDG scanning.

**Conclusion:**

We conclude that future clinical evaluation of the [^68^Ga]Ga-siderophore approach to infection imaging requires a deeper understanding of the kinetics and thermodynamics of transchelation of ^68^Ga between the siderophores and transferrin.

**Supplementary Information:**

The online version contains supplementary material available at 10.1007/s00259-026-07831-4.

## Introduction

Aortic graft infections (AGI), are associated with high morbidity and mortality [1, 2]. The range of micro-organisms underlying AGI is extremely wide and depends upon a complex interaction between multiple factors [2]. For example, early onset AGI results from contamination at the time of surgical implantation, or placement of grafts in a pre-existing infected field. Late onset AGI is generally the result of haematogenous spread from a distant site, or contiguous spread of local infection (e.g., from the gastrointestinal tract).

*Staphylococci* are the commonest group of gram-positive organisms implicated, including *Staphylococcus* aureus and coagulase-negative *staphylococci*, which are typical skin commensals. *Enterobacterales* are also commonly involved and include gram-negative organisms (e.g., *Escherichia coli*), anaerobes and *enterococci*. Polymicrobial infections with such bowel flora, especially involving *Candida* species, may herald fistula development between an abdominal graft and the gastrointestinal tract [1, 2, 4]. Explantation and vascular reconstruction can be curative but carry an associated mortality up to 30% [3]. Without surgery, mortality is between 67 and 100% even with antimicrobial therapy, demanding a high degree of certainty from diagnostic investigation [4, 5]. Despite widely accepted diagnostic criteria [6], AGI can be difficult to diagnose. Computed tomographic angiography, the first-line imaging investigation, has modest accuracy. [^18^F]fluorodeoxyglucose positron emission tomography/CT (FDG PET-CT) and leukocyte scintigraphy image the immune response to infection, rather than infection per se. FDG PET-CT has good sensitivity but lower specificity and cannot reliably distinguish infection from sterile inflammation. Leukocyte scintigraphy has high accuracy for infection but is more complex to perform requiring specialist facilities for white cell labelling and needing delayed imaging. Both modalities have been reported to show reduced sensitivity in chronic infections, in patients receiving antimicrobial therapy and in elderly and immunocompromised patients [7–9].

PET tracers targeting bacteria-specific metabolic pathways could overcome some of these limitations [10]. [^18^F]fluorodeoxysorbitol showed specific uptake in Gram-negative enterobacterial infections in humans [11] and [^11^C]para-aminobenzoic acid showed uptake in Staphylococcus aureus infections in preclinical models and favorable biodistribution in healthy volunteers [12]. Siderophores, which are a range of small molecules secreted by bacteria and fungi to help sequester essential iron (in some cases with individual species producing their own structurally unique siderophores and corresponding receptors for their iron complexes), offer another potential bacterial-specific target. Gallium (Ga^3+^) forms complexes with many siderophores that are isostructural with their Fe^3+^ complexes. Labelled with ^68^Ga, such complexes could target bacterial iron-siderophore complex receptors; pre-clinical data suggest that they can localize to infections [13–17].

**Fig. 1:**
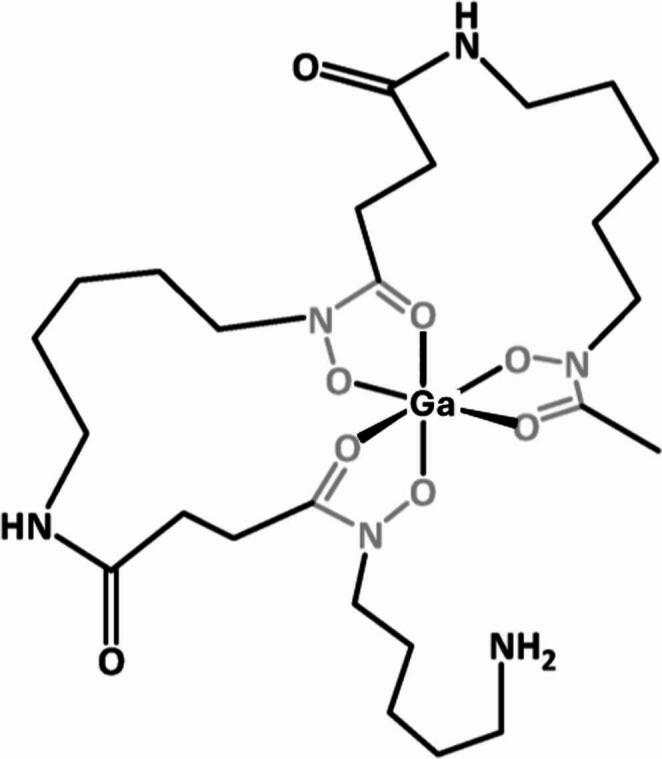
Schematic structure of gallium DFO complex, based on the assumption that the complex is isostructural with the iron(III) complex

Here we evaluate this concept using the siderophore desferrioxamine-B (DFO), chosen because its gallium complex (Fig. 1) believed to be isostructural with the iron complex [18], is taken up in bacteria of interest in AGI (*S. aureus*,* Streptomyces species*,* Pseudomonas aeruginosa and Yersinia enterocolitica*) [19] and DFO is a licensed medicine, presenting low regulatory barriers to evaluation in humans. DFO has been used clinically for treating iron overload for many years [15, 20].

We report (i) a method for good manufacturing practice (GMP) production of [^68^Ga]Ga-DFO; (ii) assessment of its biodistribution in mice; and (iii) assessment of its biodistribution, dosimetry and potential for imaging in a first-in-human PET study in patients with suspected AGI.

## Methods

Sourcing of equipment and consumables, development and characterization of [^68^Ga]Ga-DFO are described in supplemental information.

### Uptake of [^**68**^Ga]Ga-DFO on infected vascular stents in vitro.

Excess sterile stent material, comprising nitinol stents covered with polytetrafluoroethylene, from operations performed in the Department of Vascular Surgery, St Thomas’ Hospital were immersed in 9 mL brain heart infusion broth with > 10^6^ CFU (1 mL) of *S. aureus*, *E.coli*, *P. aeruginosa* or *Candida albicans*, or 1 mL of sterile phosphate buffered saline (PBS, control) in 50 mL Falcon tubes. After 24 h, stents were washed three times with sterile PBS and resuspended in 10 mL of Davies minimal broth. 50 µL of [^68^Ga]Ga-DFO (10 µg DFO, 3–4 MBq), was prepared (supplemental information) and added to each stent followed by incubation at 37 °C while shaking (70 rpm) for 30 min. Stents were washed three times with sterile PBS, gamma-counted and imaged with a phosphor imager.

### Radiolabeling including GMP Clinical Production.

Manufacture for clinical production was carried out in a licensed GMP radiopharmacy. All starting materials were from authorised suppliers using sterile GMP grade or licensed materials. Analytical methods were validated and were in line with standard operating procedures used within the facility for 68Ga-radiopharmaceuticals. Radiolabelling took place in a Grade A isolator (Amercare) by trained and qualified operators using aseptic technique, and environmental monitoring was carried out during manufacture to ensure an EU GMP Grade A environment was maintained. Process validation was carried out on three batches before starting clinical production and all results were in line with the product specification. For manufacture of patient doses and during validation, a vial of desferrioxamine (DFO, desferrioxamine mesylate, 500 mg, Novartis) was reconstituted with water for injection (5 mL). 0.1 mL of the solution was diluted 100-fold with water for injection. The IRE GalliAd 68Ge/68Ga generator was eluted with 1.1 mL 0.1 M HCl into an Eluatic 15 mL evacuated vial using a Lectrocath line and 21G Sterican needle. 30% sodium acetate (EDTA-free, 3.6 M, pH 9, Torbay Pharmaceuticals, product code Y8247C, 60 µL) and the diluted DFO (0.1 mL) was added. Radiochemical purity was evaluated by iTLC (mobile phase 1 M ammonium acetate in 1:1 v/v H2O and methanol, stationary phase iTLC-SG) after 10 min and after 3 h (for stability assay – storage at ambient temperature). Sterility testing was performed by membrane filtration according to the British Pharmacopoeia method following sterility method validation. pH was measured using pH indicator strips. Appearance was assessed visually. Endotoxin testing was performed using a Charles River Endosafe^®^ PTS endotoxin tester following validation of the method. Radionuclidic purity (Ge-68 contamination) was assessed after decay of gallium-68 using a sodium iodide well counter.

### Preliminary serum stability assessment.

A sample of [^68^Ga]Ga-DFO was diluted (1:1 v/v) with either human serum (Sigma Aldrich, H4522) or PBS (control) and incubated for 60 min at 37 °C. [68Ga]Ga-acetate was treated similarly as a control. Ice-cold acetonitrile was added (1:1 v/v) then vortexed for 1 min and centrifuged (13,000 rpm for 5 min). Supernatant and precipitate were separated and gamma counted. Supernatant was analyzed by iTLC and RP-HPLC, as described above, after five-fold dilution with water.

### Lipophilicity and mass spectrometry

Measurements were performed by octanol extraction and liquid chromatography-mass spectrometry (LCMS) (electrospray ionization) as described in supplementary information.

### Preclinical PET scanning and ex vivo biodistribution.

PET/CT imaging and biodistribution experiments were performed on three healthy female Balb/C mice (6–9 weeks old, Charles River UK Ltd), Mice were anesthetized with isoflurane (3%, Animalcare, York, UK, in O_2_), injected via a tail vein with [68Ga]Ga-DFO in PBS (150–200 µL, 1.2–1.5 MBq, 0.2–0.3 µg DFO) and scanned dynamically for 60 min with a nanoScan^®^ PET/CT scanner (Mediso Medical Imaging Systems, Budapest, Hungary). Images were reconstructed using Nucline software (version 2.00) and analyzed using VivoQuant software (version 1.23); (further details in supplemental information). Immediately after scanning, mice were sacrificed for organ harvesting and counting. Explanted bladders were used to provide ex vivo urine samples for HPLC analysis (supplemental information). Extrapolation of quantitative biodistribution data from PET imaging was used to estimate human dosimetry (supplemental information).

### Human in vivo PET/CT scanning.

Two patients, designated patient 1 and patient 2, over 18 years with high suspicion of AGI and a standard-of-care [^18^F]FDG PET/CT scan showing uptake in the graft, were recruited from the Vascular Surgery Department at St Thomas’ Hospital (clinicaltrials.gov/NCT05285072). Patients receiving supplemental iron within 1 week of the research scan were excluded. Their clinical histories are described in supplemental information.

Patient 1 was scanned five days and patient 2 seven days after positive FDG scans. Each patient was administered [^68^Ga]GaDFO (177 and 171 MBq, respectively) and scanned using one of two protocols, (Figure S1). In protocol A, blood radioactivity was measured by scanning the aorta. Blood and plasma activities thus determined were fitted to a bi-exponential function. In Protocol B, venous blood was sampled at intervals for counting and speciation analysis, and urine was collected 90 min after injection for counting (supplemental information). Patients were contacted 24 h later to assess any concerns.

### Speciation analysis of ^68^Ga in plasma by size exclusion chromatography

Speciation of [68Ga] in plasma and urine from Patient 2 (1 mL) was analyzed using PD-10 desalting columns (supplemental information), eluting with PBS in 1 mL fractions. In addition, human plasma samples were incubated with [68Ga]Ga-DFO with various concentrations (0.03, 0.3, 3, 30, 700 µg/mL) of DFO in vitro in the presence of NaHCO_3_ (20 mM) at 37 °C for 5 min and analyzed similarly.

## Results

### Radiolabeling and radiochemical analysis

Method development for production of [^68^Ga]Ga-DFO and its radiochemical characterization are described in supplementary information (Figs. S2-S8). HPLC of [^68^Ga]Ga-DFO produced with acetate buffering, using the TFA-free gradient, showed a single radioactive species (Fig. S5C), whose mass spectrum showed a single molecular ion with *m/z* corresponding to a 1:1 Ga-DFO complex (Fig. S7) that was hydrophilic (logD − 2.9 ± 0.4) in > 95% radiochemical yield as determined by iTLC (Fig. S8). Endotoxin testing gave < 175 EU/dose and sterility testing showed no growth. The pH of the product was 4.4 and the final concentration of DFO was 79 µg/mL. All results during process validation and clinical production were in line with the product specification.

### Serum and urine stability in vitro

Analysis of the stability of [^68^Ga]Ga-DFO in human serum by protein precipitation with acetonitrile, followed by centrifugation and HPLC analysis of the supernatant, showed only [^68^Ga]Ga-DFO with no significant transchelation of ^68^Ga by serum proteins, as detailed in supplemental information. After incubation of [^68^Ga]Ga-DFO and [^68^Ga]Ga-acetate with human urine for 30 min, radioHPLC showed that both are chemically altered (supplemental information).

### Uptake on infected vascular stents

**Fig. 2:**
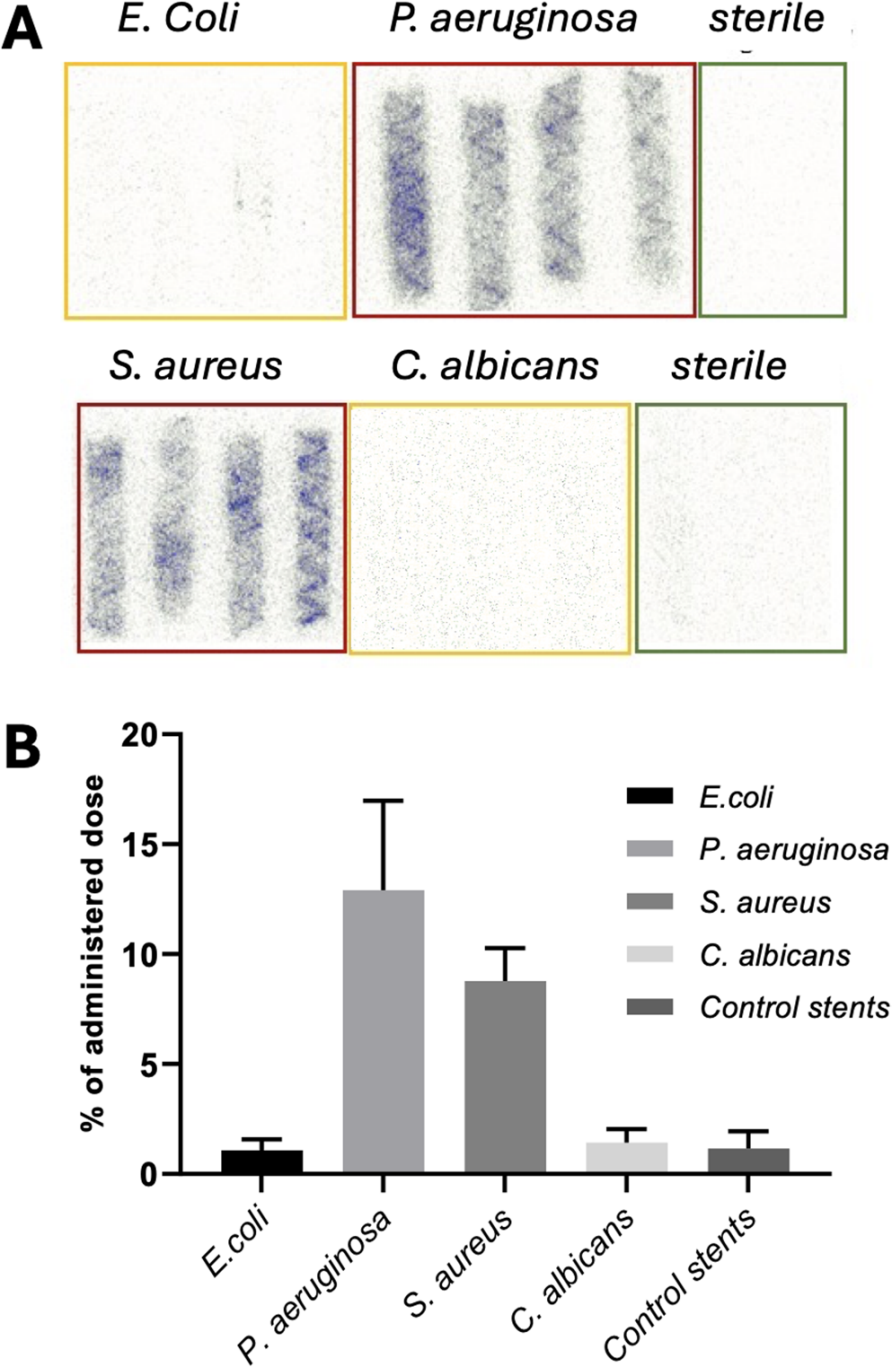
Phosphorimager images (**A**) and % administered dose bound (**B**, mean ± SD, *n* = 4) showing [^68^Ga]Ga-DFO uptake in infected stents and controls

After incubation with artificially infected vascular stents, [^68^Ga]Ga-DFO showed highest uptake in *S. aureus* (8.77 ± 1.5% administered dose (% AD)) and *P. aeruginosa* (12.9 ± 4.07% AD), while uptake in *E. coli* and *C. albicans* and controls was negligible (< 1.4%), demonstrating species-specific uptake (Fig. 2).

### Preclinical in vivo study.

**Fig. 3:**
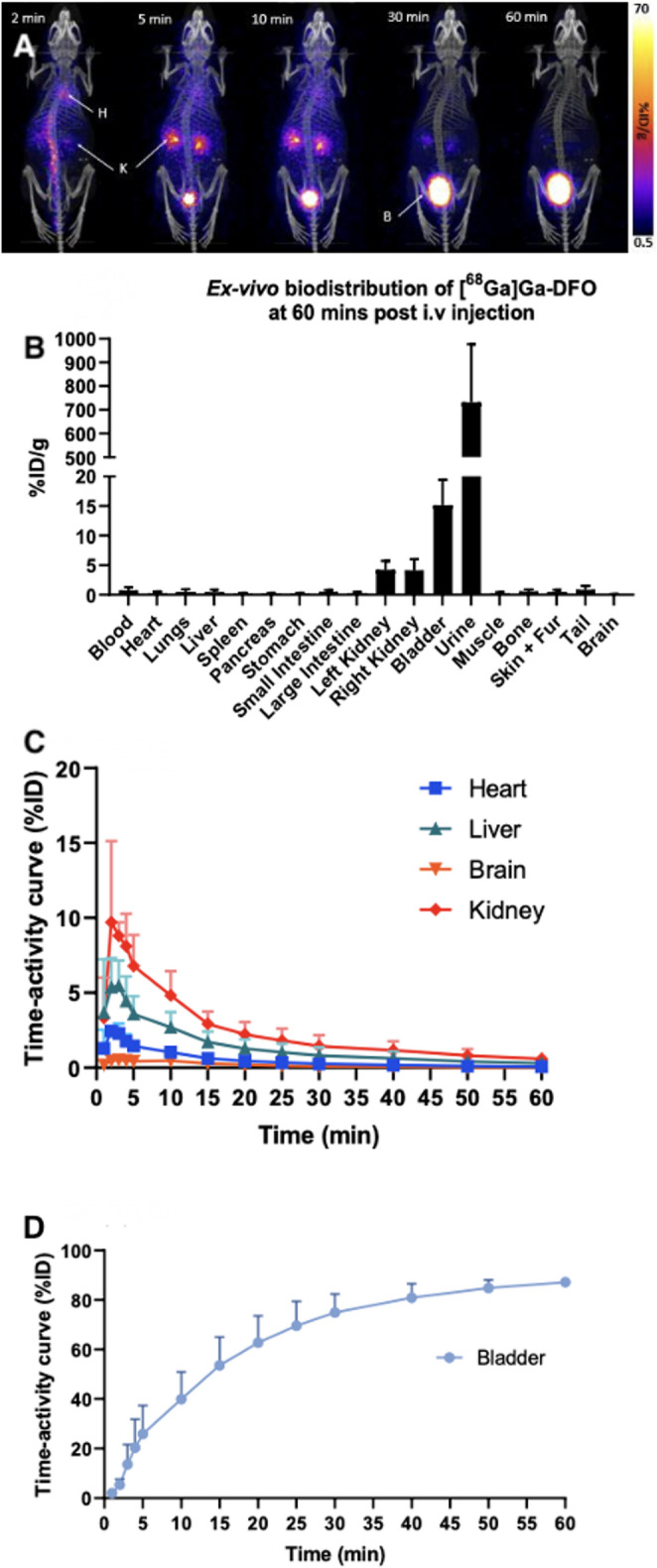
**A**: Exemplar PET/CT MIP images of [^68^Ga]Ga-DFO distribution in a healthy Balb/C mouse; H: Heart, K: Kidney, B: Bladder. **B**: ex vivo biodistribution at 60 min post injection, mean ± SD. **C**: Time-activity curves of heart, liver, brain and kidney, derived from PET images. **D**: Time-activity curve for bladder (mean ± SD)

PET/CT images of [^68^Ga]Ga-DFO distribution in healthy mice showed rapid clearance from blood, rapid renal excretion and no significant uptake in other organs; almost all activity (90%) was in urine by 60 min (Fig. 3). Ex vivo biodistribution at 60 min was consistent with images, with 732 ± 245%ID/g in the urine. RP-HPLC of urine revealed high and low abundance peaks at 2 and 17 min, indicating conversion into chemical forms other than [^68^Ga]Ga-DFO (Fig. S11).

When extrapolated to the human phantom, the pre-clinical data indicated an effective dose of 0.025 mSv (supplemental table S1).

### Human in vivo study.

**Fig. 4:**
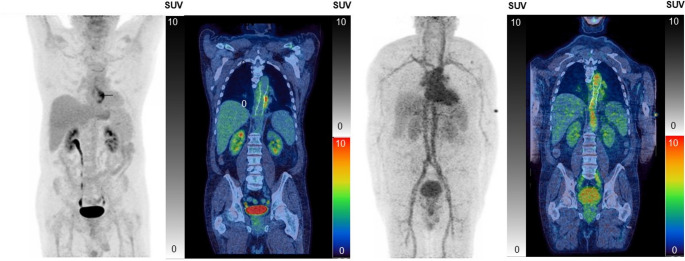
Patient 1: PET/CT scans. FDG maximum intensity projection (MIP) and coronal fused FDG images at 60 min p.i. showing focal uptake related to the aortic graft (left two panels); [^68^Ga]Ga-DFO MIP, showing high vascular uptake and coronal fused [^68^Ga]Ga-DFO without specific tracer uptake in the aortic graft at 90 min p.i. (right two panels)

**Fig. 5:**
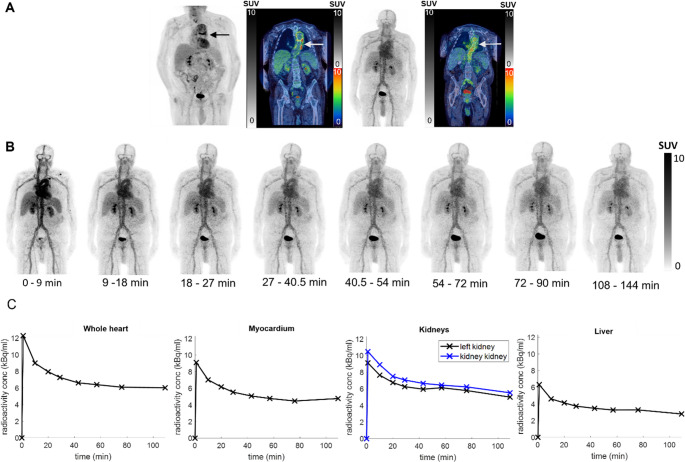
Patient 2: PET/CT scans and associated data. A: FDG MIP and coronal fused FDG images showing focal FDG uptake related to the aortic graft 60 min p.i. (left panels); [^68^Ga]Ga-DFO PET MIP showing high vascular uptake, and coronal fused [^68^Ga]Ga-DFO PET-CT without specific tracer uptake in the aortic graft 90 min p.i. (right panels); B: serial MIPs showing [^68^Ga]Ga-DFO over time; C: Decay-corrected time-activity curves of heart, kidneys and liver; curves for other organs are shown in Fig. S12

No adverse or clinically detectable pharmacologic effects, significant changes in vital signs, laboratory results or electrocardiograms were observed post-scan in either patient. Neither patient reported any concerns 24 h later. No specific [^68^Ga]Ga-DFO uptake was observed in the patient grafts (Figs. 4 and 5).

Time-activity curves for the heart, kidneys and liver (which, with spleen, showed highest uptake) are shown for Patient 2 in Fig. 5C. Uptake in all organs (figure S12) peaked during the first 9 min. Red marrow (0.008 mSv/MBq) and urinary bladder wall (0.005 mSv/MBq) received the highest radiation doses. The overall effective dose was estimated as 0.024 mSv/MBq (4.8 mSv from 200 MBq of injected radioactivity, (table S2).

**Fig. 6:**
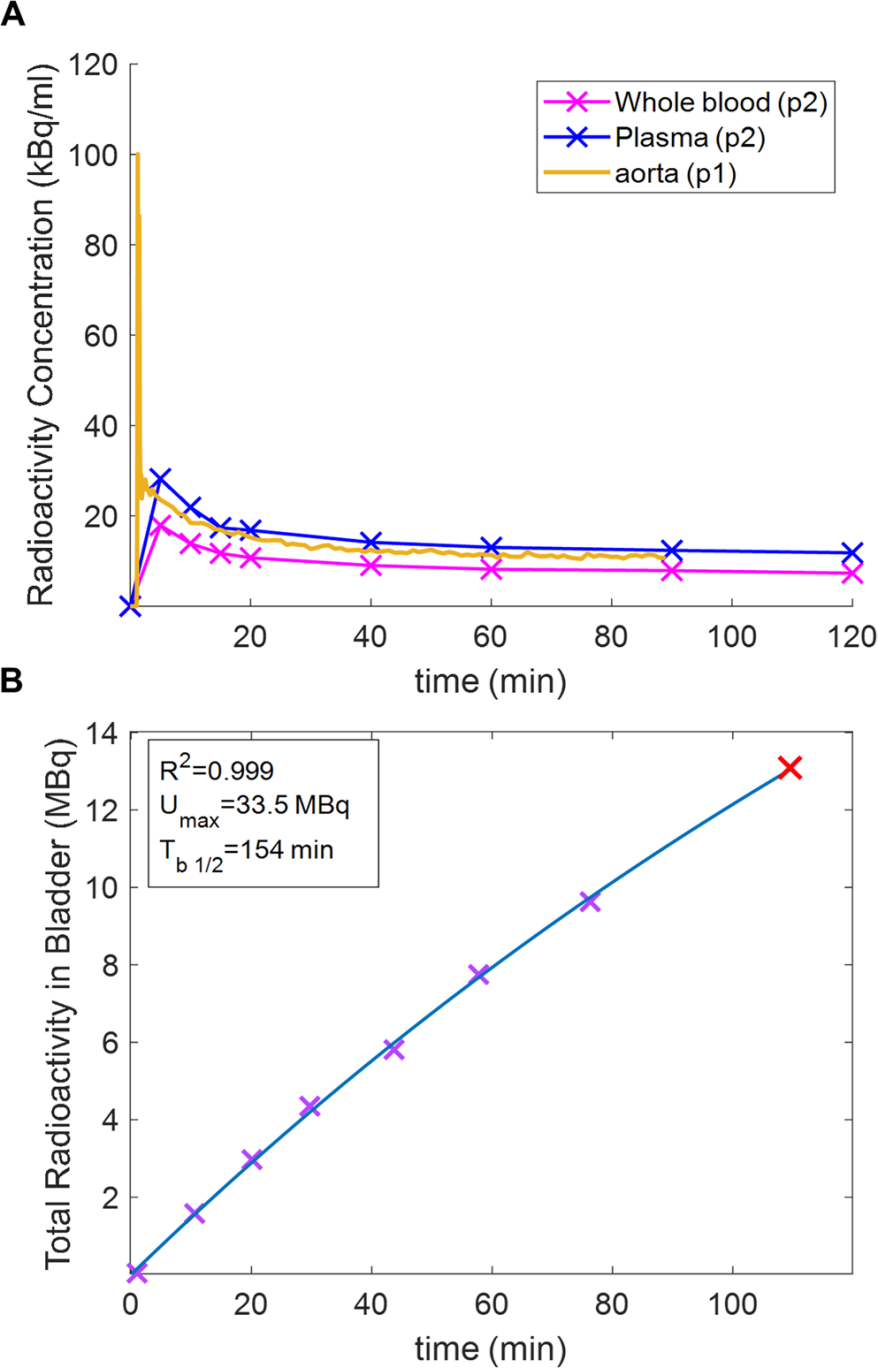
**A**: Radioactivity concentration from whole blood and plasma in patient 2 (pink, blue) and from quantitative PET imaging of the aorta (gold) in patient 1. **B**: Radioactivity measured in bladder from PET images (purple crosses) and voided urine (red cross) for patient 2 and its fitted curve

Both patients initially showed rapid renal excretion of ^68^Ga, but unlike mice, where renal excretion approached completion, the human subjects showed high vascular uptake persisting 2 h p.i. (Figures 4, 5 and 6). The blood half-life of [^68^Ga]Ga-DFO was biphasic with long half-lives of 348 and 320 min (coefficients 9.70 and 12.85) and short half-lives of 7.7 and 5.1 min (coefficients 15.32 and 16.97) for patients 1 and 2, respectively (Table S3); assuming 5.4% of the body is blood, 24% and 16%, respectively, of injected activity remained in blood after 2 h in patients 1 and 2 (cf. <2% in mice at 1 h). Urine samples from patient 2 were fitted to an exponential to determine the maximum accumulated urine activity (U_max_) and biological half-life (T_b1/2_) (Fig. 6).

### Revisiting serum stability [^68^Ga]Ga-DFO

**Fig. 7:**
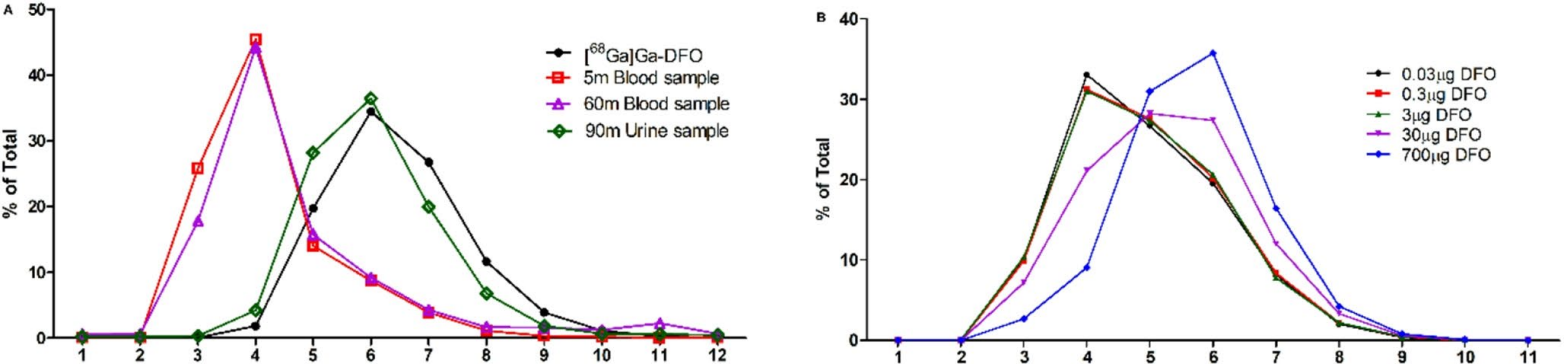
(**A**) Percentage of radioactivity in fractions collected from PD10 column analysis of [^68^Ga]Ga-DFO, blood samples collected at 5 min, 60 min and urine sample collected at 90 min after injecting [^68^Ga]Ga-DFO (patient 2); (**B**) Percentage of activity in fractions collected from PD10 column analysis of [^68^Ga]Ga-DFO and human serum incubation mixture with 0.03, 0.3, 3, 30 and 700 mg/mL DFO

The discrepancy between the rapid and almost complete renal excretion in mice and the prolonged vascular retention in humans prompted deeper analysis of the behavior of [^68^Ga]Ga-DFO in human blood. Size-exclusion chromatography of clinical ex vivo plasma (5 and 60 min post-injection of [^68^Ga]Ga-DFO, Patient 2) and urine samples (90 min post-injection) is shown in Fig. 7A. Whereas [^68^Ga]Ga-DFO in pure control samples elutes mainly in fraction 6, consistent with a low-molecular weight species, in the plasma samples, by 5 min p.i., fraction 4 was the most radioactive. This pattern was unchanged by 60 min p.i. Thus, in plasma in vivo, even by 5 min p.i., almost all the radioactivity was protein-bound.

**Fig. 8:**
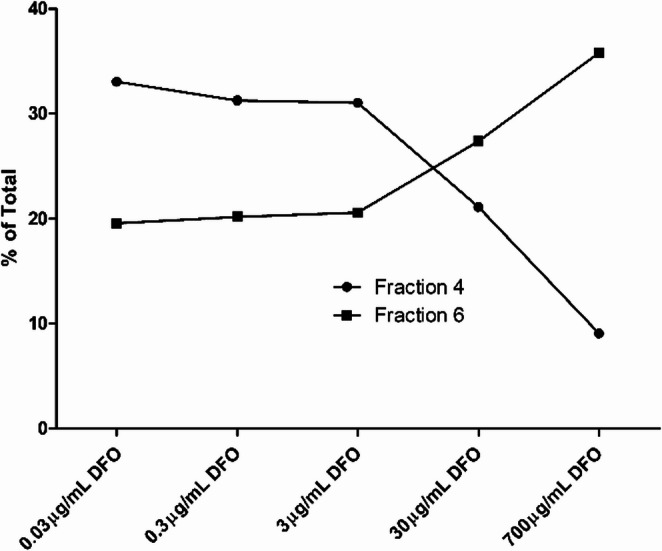
Percentage of activity in fractions 4 and 6 collected from PD10 column analysis of [^68^Ga]Ga-DFO and human serum incubation mixture with 0.03, 0.3, 3, 30 and 700 mg/mL DFO

To investigate the effect of DFO concentration on the extent of protein binding in plasma, [^68^Ga]Ga-DFO (low activity to minimize baseline DFO concentration) was incubated in vitro with donated human plasma containing increasing amounts of unlabeled DFO. Increasing DFO concentration resulted in more low-molecular weight ^68^Ga (fraction 6, Figs. 7B and 8); in plasma with very low DFO concentration, there was rapid and extensive binding of ^68^Ga to protein, which was increasingly delayed or prevented as the additional DFO concentration increased.

## Discussion

Although humans have high tolerance for DFO (max. 6 g/day i.v.), this preliminary study aimed to formulate [^68^Ga]Ga-DFO using the minimum amount of DFO required to achieve high radiochemical yield and purity for clinical use, via a simple radiolabeling procedure. A kit containing sodium bicarbonate to neutralize the hydrochloric acid in the ^68^Ga generator eluate (similar to the prostate cancer tracer, Galliprost ([^68^Ga]Ga-THP-PSMA) [21]was successfully produced for preclinical evaluation. However, the presence of EDTA in commercially available GMP sodium bicarbonate in the clinical formulation prevented efficient chelation of ^68^Ga by DFO, and the cost of bespoke EDTA-free GMP sodium bicarbonate was prohibitive. The alternative published method [19], using sodium acetate buffer, produced GMP-quality [^68^Ga]Ga-DFO suitable for first-in-human use. A single radioactive species was produced with high radiochemical purity (rendering post-labeling purification unnecessary), low lipophilicity and a mass spectrum consistent with a 1:1 complex between Ga^3+^ and DFO analogous to the Fe^3+^ complex, giving confidence that iTLC was sufficient for clinical release.

The efficient uptake of [^68^Ga]Ga-DFO in *S. aureus* and *P. aeruginosa*, but not in *E. coli* or *C. albicans*, cultured on stent samples, was consistent with published species-specific uptake in these bacteria [19]. [^68^Ga]Ga-DFO showed resistance to protein binding/transchelation in our preliminary in vitro serum stability studies, as previously reported [19]. These characteristics encouraged in vivo PET imaging in healthy mice, where the rapid clearance from blood via renal excretion was consistent with both published animal studies [19] and expectation for a hydrophilic small molecule, suggesting no significant protein binding.

These pharmacokinetic attributes, and the acceptable predicted radiation dosimetry estimates generated (table S1), encouraged human evaluation. Published animal work demonstrating [^111^In]In-DFO targeting *S. aureus*-induced abscesses [22] and [^68^Ga]Ga-DFO targeting *S. aureus* and *P. aeruginosa* infections [19] also supported this approach. The biodistribution in humans with established aortic graft infections, however, was unexpectedly different. Radioactivity was excreted renally but much more slowly than in mice, with high vascular retention and moderate uptake in liver, spleen and kidneys - organs with high vascular density - reminiscent of ^68^Ga and ^67^Ga tracers that bind to transferrin, such as ^68^Ga-citrate [23].

High vascular retention in humans, evident qualitatively from the prominent vascular activity on the PET images and the substantial longer half-life component of the biexponential blood clearance model, could confound the [^68^Ga]Ga-siderophore approach to infection imaging and therefore required further investigation. The extent of this problem can be tentatively inferred from coefficients of the terms in the biexponential blood activity model (Table S3), which suggest about 40% of the blood radioactivity (43% patient 1, 39% patient 2) clears with a long half-life attributable to a protein-bound fraction, whereas in mice there is no significant long half-life component. Size exclusion chromatography of plasma samples from patient 2 at 5 and 60 min p.i. showed that most of the ^68^Ga was protein-bound by 5 min after injection. This casts doubt on our initial in vitro serum stability study, using protein precipitation and HPLC, which did not mimic either the relative concentrations of DFO and apotransferrin (which is known to bind Ga^3+^ efficiently [23]) in blood in the human in vivo studies, or the possible in vivo transchelation involving transferrin, because efficient iron or gallium binding to transferrin requires bicarbonate, which is present in blood in vivo but was not used in the preliminary serum stability study. Bicarbonate was also omitted from serum stability measurements by Petrik et al. [19] Transferrin levels were not measured in the human subjects and were assumed to be within the normal range (ca. 30 µM [24]). In our preliminary serum stability experiments, this led to a transferrin-to-DFO molar ratio of around 1:0.1, or 1:0.14 for apotransferrin. In the mouse PET imaging experiments, based on estimated literature values of mouse plasma volume (1.6 mL) [25] and mouse plasma transferrin concentration (112 µM) [26], a transferrin: DFO molar ratio in plasma of 1:3 can be estimated; whereas the dose administered to the patients results in a much higher apotransferrin: DFO ratio of around 300:1.

The in vitro plasma protein binding studies conducted in the presence of bicarbonate, with a range of transferrin: DFO concentration ratios (higher and lower than estimated in the human in vivo studies) using size-exclusion chromatography showed that protein binding occurred but was suppressed at higher DFO concentrations, although not completely even at the highest DFO concentration (1065 µM, a transferrin: DFO ratio of ca. 1:35). The estimated transferrin: DFO ratio in the human PET studies (300:1) falls at the upper end of the range studied in vitro and is sufficient to induce significant transchelation of ^68^Ga. Thus, transferrin appears the most likely protein implicated in plasma protein ^68^Ga binding. We attribute the lack of protein binding in the initial serum stability experiments to the absence of bicarbonate and the low transferrin: DFO ratio employed. In the mouse in vivo study, we attribute the rapid vascular clearance, which implies minimal transferrin binding, to the relatively low transferrin: DFO concentration ratio. In humans, the much larger plasma volume, by a factor of almost 2000, provides a larger pool of transferrin, leading to a transferrin: DFO ratio 2 orders of magnitude higher than in mice. This preliminary analysis provides pointers to further research rather than a complete rationale for the pharmacokinetic difference between humans and mice. Nevertheless, it highlights a potential major pitfall for the wider ^68^Ga-siderophore approach to infection imaging.

The current diagnostic ‘gold standard’ does not require positive identification of the underlying causative pathogen to confirm an AGI case [6]. Apart from the high vascular background presumed due to transferrin binding, failure to image infection could result from (1) absent specific uptake by certain organisms, (2) failure of the tracer to penetrate bacterial biofilms protecting organisms adherent to the graft surface (3) micro-organisms present in low density e.g., prior antimicrobial therapy and (4) prolonged treatment with antimicrobials [27, 28]. While patient 1 had definitive microbiological evidence of a P. aeruginosa AGI, the underlying pathogen in patient 2 was not identified.

Both patients received antimicrobials and the tracer should ideally be tested as well in patients who have not received any therapy. Iron kinetics can be affected during infection, and whilst it seems unlikely that infection-related mild reduction in transferrin levels would have been sufficient to overcome transchelation of 68Ga3 + to transferrin, our data suggest that in future human studies with siderophores it would be beneficial to measure both plasma transferrin and transferrin iron-saturation levels [29]. The major limitation of the study was that only two patients were investigated. The study was suspended during the pandemic, then hampered by an international shortage of gallium generators and closure of our radiopharmacy for necessary refurbishment. Following the unexpected biodistribution in humans compared to preclinical models, a finding which was unlikely to change by scanning more subjects, we prioritized reporting our findings, as they highlight the need for caution in extrapolating preclinical investigations of [^68^Ga]Ga-DFO and other ^68^Ga-siderophores (such as [^68^Ga]Ga-triacetylfusarinine C (TAFC), [ ^68^Ga]Ga-ferrioxamine-E (FOXE) [30] and [^68^Ga]Ga-schizokinen [31], to the human in vivo context.

## Conclusion

The pharmacokinetics of [^68^Ga]Ga-DFO in humans and mice differ dramatically, showing greatly increased protein binding (most likely to transferrin) in human plasma, which has a much larger transferrin pool due to the approximately 2000-fold difference in blood volume. Despite only 2 patients being scanned, this finding was supported by subsequent experiments and strongly suggests that [^68^Ga]Ga-DFO is susceptible to transchelation with transferrin, which is more extensive in humans because the transferrin: DFO concentration ratios are higher than in mice. This is a potentially confounding factor for the ^68^Ga-siderophore approach to infection imaging, and the kinetics and thermodynamics of the transchelation between siderophores and transferrin need further research. If the transchelation hypothesis is supported by further experiments, the problem could potentially be addressed by using siderophores that are kinetically more resistant to transchelation, or in the case of [^68^Ga]Ga-DFO, by administering it with much larger doses of DFO. This is feasible because DFO is licensed for administration in relatively large doses. Other microbial siderophores do not yet offer this advantage. The 68Ga-siderophore approach to infection imaging requires deeper understanding of the kinetics and thermodynamics of transferrin transchelation.

## Supplementary Information

Below is the link to the electronic supplementary material.

Supplementary Material 1

Supplementary Material 2

## Data Availability

The datasets generated during and/or analysed during the current study are available from the corresponding author on reasonable request.
