# Supplementary material for: The 68Ga-siderophore approach to infection imaging: evaluation of [68Ga]Ga-DFO in patients with vascular graft infection
Source: Eur J Nucl Med Mol Imaging. 2026 Mar 6;53(6):4151–61. doi: 10.1007/s00259-026-07831-4 (PMC13121275; doi:10.1007/s00259-026-07831-4)
Supplement: Supplementary file 1 — Supplementary Material 1 [file 259_2026_7831_MOESM1_ESM.docx]

**The ^68^Ga-siderophore approach to infection imaging: evaluation of [^68^Ga]Ga-DFO in patients with vascular graft infection**

Submitted to European Journal of Nuclear Medicine and Molecular imaging

Sally F Barrington^1^, Margaret Cooper^1^, Georgios Krokos^1^, Zilin Yu^1^, Afnan MF Darwesh^1,2^, Victoria Gibson^3^, Ronan Tegala^3^, Julia E Blower^1^, Maria Dibua^1^, Armidita Jacob^1^, Joemon John^1^, Sofia Pereira^1^, Laura Cole^4^, Nicholas Price^5^, Morad Sallam^4^, Philip J Blower.^1^

**^1^** King’s College London and Guy’s and St Thomas’ PET Centre, School of Biomedical Engineering and Imaging Sciences, King’s College London, King’s Health Partners, London, UK, ^2^ Department of Radiologic Sciences, Faculty of Applied Medical Sciences, King Abdulaziz University, Jeddah, Saudi Arabia, ^3^ Department of Nuclear Medicine, ^4^Department of Vascular Surgery and ^5^Directorate of Infection, Guy's & St Thomas' NHS Foundation Trust, London, UK.

Corresponding author

Sally Barrington

School of Biomedical Engineering and Imaging Sciences

St Thomas’ Hospital

London SE1 7EH

+44 207188 4988

[sally.barrington@kcl.ac.uk](mailto:sally.barrington@kcl.ac.uk)

**Supplementary information**

**Methods**

All reagents and consumables were from Sigma Aldrich and Thermo Fisher Scientific unless specified otherwise. DFO was obtained from Novartis (Desferal 500 mg vial) and good manufacturing practice (GMP) grade sodium acetate from Torbay Pharmaceuticals (product code Y8247C). For preclinical studies, 68Ga was obtained from a 68Ge/68Ga generator (Eckert & Ziegler) by eluting with 5 ml of 0.1 M ultrapure HCl (ABX) and collecting 5 x 1 ml fractions. For clinical studies 68Ga was obtained from a Galli Ad® (IRE) generator in 1.1 mL 0.1M HCl. Instant thin layer chromatography (iTLC) was performed using Agilent technologies silica gel-impregnated glass microfiber strips (10 cm length), scanned by a LabLogic mini scan TLC reader with positron (β+) detector and analysed with Laura software. High-performance liquid chromatography (HPLC) was implemented using an Agilent Eclipse XDB C18 5 μm 4.6 × 150 mm reversed phase (RP) column and Agilent technologies 1200 series with in-line ultraviolet (UV) detection (220 nm) with Gina StarTM software version 5.8. Gradient: (0–2 min: 0 % B, 2– 24 min: 60 % B, 24–26 min: 60 % B, 26–28: min: 0%). . Liquid chromatography-mass spectrometry (LC/MS) was acquired using an Agilent Eclipse XDB C18 5 μm 4.6 × 150 mm RP column on an Agilent Liquid Chromatograph (1200 Series) with UV detection at 230 nm connected with Advion Expression LCMS mass spectrometer with electrospray ionisation source. Analysis of the mass spectra was performed with Advion Mass Express software (version, 6.4.16.1). Gamma counting was performed with an LKB Wallac 1282 CompuGamma Gamma Counter. Phosphor imaging of stents and iTLC sheets was performed using a Cyclone Plus Phosphor Imager (PerkinElmer).

**Octanol extraction/ log*D7.4* (octanol/PBS)**

Determination of lipophilicity was performed by the shake-flask method. 0.3–0.5 MBq of the [68Ga]Ga-DFO and [68Ga]Ga-acetate mixtures was added to a pre-equilibrated mixture of equal volumes of octanol and PBS (500 μl/500μl). Samples were vortexed for 2 minutes with a Multi Vortex Mixer V-32 (Grant Bio) to obtain a good separation between the two layers. A sample (200 μL) was taken from each layer and analysed with a gamma counter.

**LC/MS of ^nat^Ga-DFO and [^68^Ga]Ga-DFO**

natGa-DFO was prepared by mixing an aqueous solution of GMP-grade DFO (10 mM), GMP- grade sodium acetate (3.6 M) and an aqueous solution of gallium nitrate (10 mM) (Thermo Fisher Scientific 32116). The mixture was diluted 10-fold with water to bring the final concentration to 1 mM. 20 μL of [68Ga]Ga-DFO (2 MBq) and natGa-DFO were used for radioLC/MS analysis.

**Mouse PET/CT analysis**

PET/CT static images were generated from the dynamic data and reconstructed using 1-minute bins (0-5 minutes), 5-minute bins (5-30 minutes) and 10-minute bins (30-60 minutes). Regions of interest (ROI) were delineated on PET/CT images for quantification of activity in selected organs and displayed as time-activity curves (TACs). The time integrated activity coefficients (TIACs) were estimated by fitting the TACs to bi-exponential curves and using the trapezoidal method. The average values for all 3 mice were used for each organ.

**Mouse biodistribution and urine sampling**

Organs taken from mice sacrificed 60 min post-injection were blotted dry, weighed and counted in a gamma counter. To assess the stability of [68Ga]Ga-DFO in urine, a 100 μL urine sample from the excised bladder was injected directly into HPLC for analysis.

**Mouse-to-human dosimetry extrapolation**

The TIACs obtained by PET imaging as described above were then extrapolated to the human 73.7 kg phantom using the following formula to give human TIACs which in turn were used as input in OLINDA/EXM 1.1 to estimate the delivered dose.

$${TIAC}_{human}={TIAC}_{mouse}\cdot\frac{m_{human}/M_{human}}{m_{mouse}/M_{mouse}}$$

where m_human_and m_mouse_are the weights of the corresponding organs for the human phantom and the mice, respectively, and M_human_and M_mouse_ are the total weights for the human phantom and the mouse, respectively.

**Stability in human urine**

To further investigate stability, [68Ga]Ga-DFO was incubated in human urine (Lee BioSolutions, 991-03-P) *in vitro* to assess whether [68Ga]Ga-DFO metabolism occurs in serum prior to reaching urine or in the urine. [68Ga]Ga-DFO was diluted (1:1 v/v) with either human urine or PBS and incubated for 60 minutes. [68Ga]Ga-acetate (control) incubated with human urine for 30 minutes. After incubation and before injecting into the HPLC the mixtures were diluted with water (1:1 v/v), centrifuged for 10 minutes at 13,000 rpm and passed through a MF-Millipore membrane filter (33 mm with 0.45 μm pore size, Merck Millipore, SLHA033SS) to remove any particles. 100 μL of the filtered sample (0.4–0.8 MBq) was injected into the HPLC and analysed with a mobile phase and gradient as described above.

**Patient clinical histories**

Patient 1 (male, aged 64 years) presented with intermittent haematemesis and dark stools. Investigations revealed a 9 mm mycotic aneurysm and an aorto-oesophageal fistula, requiring a thoracic aortic endovascular stent and intravenous antimicrobial treatment. C-reactive protein continued to increase post-operatively, with Pseudomonas aeruginosa x 1 and Enterococcus species x 2 isolated from blood cultures. An FDG PET-CT scan performed 7 weeks post-stenting revealed high grade focal uptake associated with the aortic graft, with maximum standardised uptake value (SUV) of 8.7 and extraluminal gas anterior to the graft. The patient underwent explant of the infected graft and aortic reconstruction 5 months after stenting, with P. aeruginosa and Enterobacter species cultured from intra-operative tissue samples.

Patient 2 (male, aged 71 years) presented with a discharging sternal wound after re-do surgery for an aneurysmal thoracic aortic dissection with a frozen elephant trunk procedure. CT angiography suggested graft infection with abnormal superior mediastinal soft tissue, enhancement of the aortic wall and air in the sac surrounding the aorta. Despite intravenous antimicrobial treatment, an FDG PET-CT scan performed 6 months post operatively suggested ongoing AGI with high grade focal and diffuse uptake associated within the graft (maximum SUV 13.1) and mediastinal soft tissue. The patient underwent explant of the graft in the descending aorta as the first part of a two-stage procedure. The patient underwent explant of the graft in the descending aorta as the first part of a two-stage procedure. No organisms from intra-operative samples were isolated.

**Human PET-CT scanning protocol**

Human images were acquired using a Discovery 710 PET-CT scanner (General Electric Healthcare, Waukesha WI, USA).


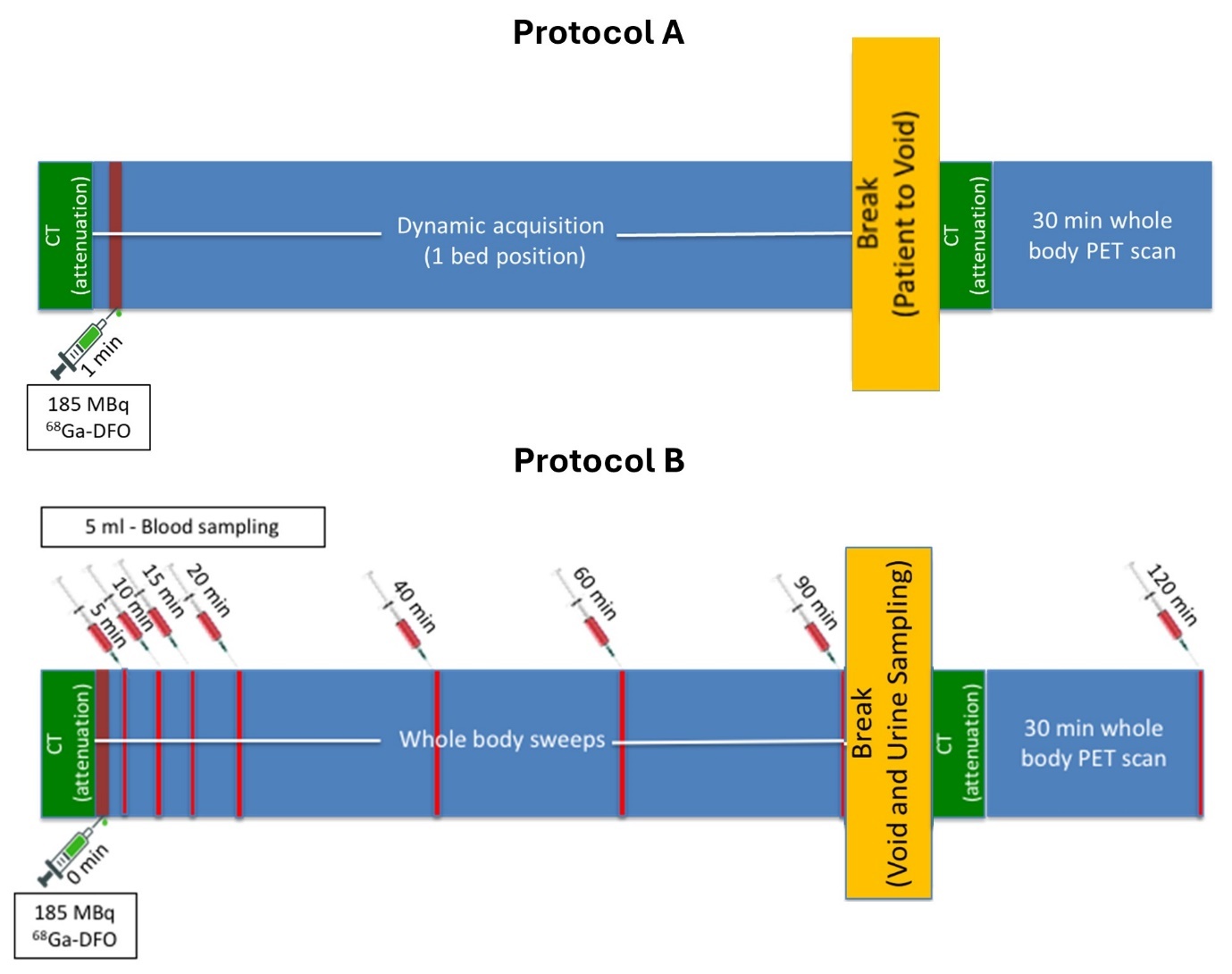


**Figure S1**: Scanning Protocols for patients undergoing PET-CT; protocol A to determine the optimum uptake time, protocol B with blood and urine sampling for estimating the biodistribution of the tracer. Red syringes indicate 5-ml blood sampling and green syringes administration of ^68^Ga-DFO. Both protocols were followed by a whole-body static PET-CT scan.

Protocol A, intended to estimate the optimum uptake time, consisted of a 90 minute dynamic scan over the infection site reconstructed using the following frame definition: 1 x 60, 12 x 5, 6 x 10, 3 x 20, 86 x 60 s. Protocol B, intended to estimate the tracer biodistribution, consisted of whole-body sweeps (3x1, 2x1.5, 2x2 min/bed position) over 9 bed positions for the first 90 minutes covering the vertex to upper thighs. Both protocols were followed by a 30-minute half-body scan at 4 minutes/bed position. All PET images were reconstructed with an ordered subset expectation maximization algorithm using 2 iterations and 24 subsets and including time of flight. The images were reconstructed in a 256 x 256 matrix and a post-reconstruction Gaussian filter of 6.4 mm was applied.

A low dose CT scan (CT parameters: 140 kVp, 65 mAs, collimation = 40 mm, pitch = 1.375) was acquired for attenuation correction with a combined estimated effective dose of 16 mSv.

All body sweeps were rigidly registered to the final 30 min scan while a summed image of the last 10 min of the dynamic acquisition in protocol B was used as the reference to register the rest of the frames using MIM software (MIM Software Inc., USA). Organs of interest were manually delineated by an experienced clinician using both CT and PET images. A volume of interest was drawn on the thigh muscle and the total muscle volume was determined by age- and gender-specific tables (https://journals.physiology.org/doi/epdf/10.1152/jappl.2000.89.1.81). The regions were then transferred to all coregistered PET images except for the bladder, for which a 20% threshold was applied on the PET images from every sweep to outline the region. The TIACs for all organs were estimated using the net activity and the organ volume, assuming physical decay after 2 h and using the trapezoidal method (<https://www.ncbi.nlm.nih.gov/pmc/articles/PMC8978196/>). These were then normalized to a 73.7 kg male phantom as defined in ICRP publication 89 (<https://www.sciencedirect.com/science/article/pii/S0146645303000022?casa_token=A421ImtLm-cAAAAA:dSBhAqx_QV3WALMeTTeqBFsXvfyzDRZmyaKo5dLwm7rQQ2JiMjFEionyl9LaOTtlnIDhQuBjyg>). The weight of each organ was estimated using its volume and organ density tables (https://itis.swiss/virtual-population/tissue-properties/database/density/). The averaged radioactivity concentration from the blood samples was used to estimate the radioactivity concentration in red marrow assuming that 5.4% of the reference phantom mass was blood.

The total activity from the urinary bladder estimated from the PET images and the collected urine (U(t)) was used to estimate the maximum accumulated urine activity (U_max_) and the biological half-life in the body (T_b1/2_ ) by fitting the following formula to an exponential curve.

$$U\left( t \right)= U_{max}\left( 1 -e^{-\frac{ln(2)}{T_{b1/2}}t} \right)$$

All dosimetry calculations were performed in OLINDA/EXM 1.1 in which a voiding bladder model was defined assuming a 3.5-h voiding interval. The remaining radioactivity was assumed to be uniformly distributed in the rest of the organs.

In protocol A venous blood was collected at 0, 5, 10, 20, 40, 60, 90 and 120 minutes. Three 0.2 mL whole-blood and three 0.2 mL plasma aliquots after centrifugation were collected from each blood sample. All aliquots were weighed and counted for 3 min on a 10-sample well counter (2470 WIZARD2; PerkinElmer), previously cross-calibrated with ^18^F to the PET scanner. The radioactivity concentration for whole-blood (C_WB_) and plasma (C_p_) was estimated by averaging the three aliquots and correcting for decay, background radiation and using the estimated scanner-to-well counter calibration factor considering the branching fraction relative difference between ^18^F and ^68^Ga.

After the whole-body sweeps, the patient scanned under protocol A voided within a container. The urine was weighed and 6 x 0.2 mL aliquots were counted on the well counter. The radioactivity concentration within urine was estimated as described earlier for blood and the average value from the 6 aliquots was used.

**Human blood sample analysis**

Each PD-10 Desalting Column was pretreated with 1 mL of 1% bovine serum albumin (BSA) to block non-specific binding and subsequently washed with 30 mL of phosphate-buffered saline (PBS). Plasma or urine samples (1 mL each) were loaded onto the columns, allowing them to fully enter the column bed. Elution was performed with 12 successive 1 mL aliquots of PBS, and each fraction was collected individually and counted with a gamma counter.

**Results**

**Development, radiolabelling and quality control for GMP Clinical Production**

Combining ^68^Ga-generator eluate with DFO and reagent grade sodium bicarbonate buffer (the latter to neutralize HCl and produce a product with a pH suitable for injection) gave a product showing a single significant radiochemical species as measured by iTLC (Rf = 0.7-0.9, c.f. unmodified [^68^Ga]GaCl_3_ Rf = 0; Fig. S2).

**Figure S2**. iTLC of [^68^Ga]Ga-DFO (**A**) and unchelated ^68^Ga (**B**) prepared with reagent grade (EDTA-free) sodium bicarbonate buffer. These chromatograms shows that [^68^Ga]Ga-DFO moved to the solvent front while unchelated ^68^Ga remained in the origin, and that the [^68^Ga]Ga-DFO produced by this method was uncontaminated by unchelated ^68^Ga. iTLC mobile phase: 1M ammonium acetate in 1:1 v/v H_2_O and methanol (pH=7).

RadioHPLC of the product showed a dominant peak with an elution time of 11 min (Fig. S3A, blue line), corresponding to the formation of [^68^Ga]Ga-DFO (as unequivocally identified by LCMS, see below), accompanied by a low level (<10%) early-eluting broad peak (3-6 min) which proved to be a chromatographic artefact (discussed below). (Under the same HPLC conditions, DFO itself, detected by its UV absorbance, eluted at 12 min (Fig. S3D)). Unfortunately, when repeating this procedure with commercially-available sterile GMP-grade sodium bicarbonate, these radioanalytical data could not be reproduced; radioHPLC showed a main peak (>97% eluted activity) at 2 min, with <3% eluting at 11 min corresponding to [^68^Ga]Ga-DFO (Fig. S3A, black line). The proprietary GMP-grade sodium carbonate used here, like others available, contains EDTA which is a known effective chelator for gallium. To determine whether the preferential complexation of ^68^Ga^3+^ by EDTA rather than DFO could be the cause of the changed HPLC behaviour, the radioHPLC was compared with that of a [^68^Ga]Ga-EDTA solution; the latter also produced a dominant peak (100% activity) eluting at 2 min, suggesting that this indeed is the case (Fig. S3C, tan and blue lines).

**Figure** **S3**. **A**: Reverse phase radioHPLC of [^68^Ga]Ga-DFO prepared with standard DFO concentration in reagent grade sodium bicarbonate (blue line) and in proprietary GMP grade sodium bicarbonate which contains EDTA (black line). In the absence of EDTA (reagent grade bicarbonate, blue) a dominant radioactive peaks at 11 min is observed, corresponding to [^68^Ga]Ga-DFO. In GMP grade bicarbonate, the dominant peak elutes at 2 min, with only a minor peak (<3% activity) at 11 min. **B**: RadioHPLC of [^68^Ga]Ga-DFO prepared with 30-fold higher DFO concentration (attempting to suppress EDTA-complex formation) in reagent grade sodium bicarbonate (blue line) and in proprietary GMP grade sodium bicarbonate which contains EDTA (black line). The formation of ^68^Ga-EDTA complex is diminished and that of [^68^Ga]Ga-DFO enhanced, but the [^68^Ga]Ga-DFO yield still does not exceed 55%. **C**: Control radioHPLC showing that ^68^Ga-EDTA complex formed in both GMP bicarbonate buffer (tan) and in EDTA solution (blue) has an elution time of 2 min, similar to that of unchelated ^68^Ga (black). **D**: UV HPLC chromatogram of DFO alone showing elution time of 12 min. Mobile phase: gradient comprising water (solvent A) and acetonitrile (solvent B) each containing 0.1% trifluoracetic acid.

Unchelated ^68^Ga, in the absence of either EDTA or DFO, also elutes at 2 min on HPLC (Fig. S3C, black line) so an alternative analytical method was required to distinguish between unchelated ^68^Ga and [^68^Ga]Ga-EDTA; this was provided by the iTLC method (Fig. S4) in which unchelated ^68^Ga remains at the origin while [^68^Ga]Ga-EDTA, formed either in control EDTA solution (Fig. S4A) or in the GMP bicarbonate buffer (Fig. S4B), elutes at the solvent front. Thus, we can confidently conclude that the presence of EDTA in the GMP-grade bicarbonate buffer is the cause of the production failure.

**Figure S4**. iTLC (silica gel impregnated glass microfiber strips) of ^68^Ga incubated with aqueous EDTA (**A**) for 10 min and ^68^Ga incubated with proprietary GMP grade sodium bicarbonate (**B**). Chromatogram **A** indicates that [^68^Ga]Ga-EDTA moves with the solvent front (c.f. unchelated ^68^Ga remains at the origin, see Fig. S2B). This supports the conclusion from radioHPLC that EDTA present in GMP sodium bicarbonate complexes ^68^Ga leading to the peak eluting at 2 min (Fig. S3A), albeit leaving some uncomplexed ^68^Ga at the origin of the iTLC strip. It also provides a means to distinguish [^68^Ga]Ga-EDTA from unchelated ^68^Ga, which is not possible with the radioHPLC method shown in Fig. S3C. iTLC mobile phase: 1M ammonium acetate in 1:1 v/v H_2_O and methanol (pH=7).

An attempt to overcome this preferential binding of ^68^Ga to EDTA by increasing the DFO concentration thirty-fold during the radiolabelling reaction partially suppressed formation of [^68^Ga]Ga-EDTA but the yield of [^68^Ga]Ga-DFO still did not exceed 55% (Fig. S3B). Addition of a heating step (80^o^C, 10 min) to the radiolabelling reaction in GMP bicarbonate buffer improved the yield/purity, producing ca. 90% [^68^Ga]Ga-DFO with <10% [^68^Ga]Ga-EDTA. This suggests that the preference of ^68^Ga binding to EDTA rather than DFO is kinetic rather than thermodynamic; nevertheless, since the heating step added complexity to the radiolabelling procedure without eliminating the need for purification, this method was not pursued further.

As can be seen in Fig. S3A and B, the radioHPLC of [^68^Ga]Ga-DFO initially conducted using a water-acetonitrile mobile phase gradient including 0.1% TFA produced a broad low-level (<10% radioactivity) radioactive signal around 3-6 min as well as the main product peak at ca. 11 min (Fig. S5A), possibly related to the low pH of the mobile phase. Surmising that this early-eluting

**Figure S5**. Effect of TFA on radioHPLC of [^68^Ga]Ga-DFO. **A**: [^68^Ga]Ga-DFO analysed using H_2_O/MeCN containing 0.1% TFA; a broad radioactive signal can be seen between 3 and 6 min accompanying the main [^68^Ga]Ga-DFO signal at 11 min; **B**: Incubation of [^68^Ga]Ga-DFO with 2% TFA for 10 min prior to analysis with the 0.1% TFA-containing H_2_O/MeCN mobile phase produces a major (>75% activity) new discrete radioactive signal with elution time of 2-3 min together with the [^68^Ga]Ga-DFO signal at 11 min, showing that TFA does indeed react with [^68^Ga]Ga-DFO; chromatograms **A** and **B** suggest that the early-eluting signal in **A** is indeed an artefact due to an on-column reaction of [^68^Ga]Ga-DFO with TFA; **C**: HPLC of [^68^Ga]Ga-DFO using a TFA-free mobile phase, showing that omitting TFA from the H_2_O/MeCN mobile phase eliminates the artefact at 3-6 min and shifts the [^68^Ga]Ga-DFO peaks a little earlier (ca. 8 min).

artefact was produced by an on-column reaction involving TFA, we repeated the radioHPLC after incubating the sample with 2% added TFA for 10 min. This produced a new dominant (>75% activity) radioactive species with an elution time of between 2 and 3 min, alongside the [^68^Ga]Ga-DFO peak at 11 min, confirming that [^68^Ga]Ga-DFO does indeed react with TFA to give an early eluting species (Fig. S5B). The occurrence of such a reaction on the column could account for the broad early-eluting radioactive peak. The early-elution artefact disappeared, and the main peak eluting a little earlier (ca. 8 min, Fig. S5A) when TFA was omitted from the mobile phase (Fig. S5C). A subsequent elution with EDTA-containing mobile phase eluted negligible activity, showing that the TFA-free mobile phase did not leave significant activity on the column. To avoid this potentially interference by TFA, the TFA-free HPLC method was used for all subsequent analyses, even though the elution time of [^68^Ga]Ga-DFO became slightly less reproducible under these conditions.

To avoid the issue of ^68^Ga-chelation by EDTA, a GMP method for producing [^68^Ga]Ga-DFO by performing the radiolabelling in commercially-available, EDTA-free GMP-grade sodium acetate buffer instead of sodium bicarbonate was used. This gave a product with both the same analytical and radioanalytical characteristics as the reagent-grade sodium bicarbonate product and a suitable pH for in vivo use. This method was validated (3 validation batches of 895 MBq, 645 MBq and 906 MBq) using a Galli Ad® (IRE) generator (elution volume of 1.1 mL) and also similarly with a 68Ge/68Ga generator (Eckert & Ziegler) with fractionated elution using the 1.6-2.7 mL fraction (1.1 mL) for radiolabelling. The Galli Ad® (IRE) generator gave better radiochemical purity as indicated by iTLC (97.9% ± 0.17%, c.f. 92.5% for Eckert & Ziegler) and was used for the human studies. Radiochemical purity (iTLC) remained at 97.9% (± 0.26%) 3 hours after labelling by iTLC. HPLC analysis using the TFA-free gradient consistently showed >95% radiochemical yield, supporting the use of iTLC alone for analysing radiochemical purity for release of clinical batches (Fig. S6). Endotoxin testing gave < 175 EU/dose and sterility testing showed no growth. The pH of the product was 4.4 and the final concentration of DFO was 79μg/mL

**Figure S6**. Exemplar iTLC (**A**) and radioHPLC (**B**) of [^68^Ga]Ga-DFO prepared for human i.v. use using GMP sodium acetate buffer. iTLC showed no radiochemical impurity at the origin and a pure product with Rf = 0.86. RadioHPLC showed a single species eluting at ca. 9 min. iTLC mobile phase: 1M ammonium acetate in 1:1 v/v H_2_O and methanol (pH=7). **B**: Exemplar radioHPLC of [^68^Ga]Ga-DFO. HPLC mobile phase: water/acetonitrile gradient (TFA-free).

**Liquid chromatography-mass spectrometry (LCMS)**

Repeating the above acetate-buffered radiolabelling procedure with the addition of ^nat^GaCl_3_ and including electrospray ionization in the HPLC detection modes showed that the HPLC peak assigned to [^68^Ga]Ga-DFO (Fig. S6B) was unchanged by the inclusion of carrier ^nat^Ga and produced an electrospray mass spectrum (Fig. S7A) corresponding to a single species with *m/z* = 627.1 and 629.1 (C_25_H_46_N_6_O_8_Ga^+^ with ^69^Ga and ^71^Ga respectively) corresponding to a 1:1 complex of Ga^3+^ with DFO as shown in the main manuscript, Fig. 1. No other significant species were detected in the mass spectrum of this fraction, and no other significant radioactive peaks were detected in the radio-HPLC. The small UV-active peak at 10.3 min gave a mass spectrum matching that expected for DFO (C_25_H_49_N_6_O_8_^+^, *m/z* = 561.1).

**Figure S7**. LCMS of [^68^Ga]Ga-DFO. **A**: UV chromatogram shows Ga-DFO eluting at 8.3 min, which matches the corresponding radioactive peak shown in Fig. S6B when corrected for the delay between detectors (60 s); metal-free DFO elutes at 10.3 min; the corresponding radiochromatogram is shown in Fig. S6B. **B**: electrospray mass spectrum of fraction eluting at 10.3 min, corresponding to free DFO (C_25_H_49_N_6_O_8_^+^, *m/z* = 561.1). **C**: electrospray mass spectrum of fraction eluting at 8.3 min, corresponding to free Ga-DFO complex (C_25_H_46_N_6_O_8_Ga^+^, *m/z* = 627.1 and 629.1). Mobile phase as in Fig. S6.

Both [^68^Ga]Ga-DFO prepared in this way, and [^68^Ga]Ga-acetate prepared as above but without DFO, showed similar hydrophilic properties with logD(octanol/PBS) values of -2.9 ± 0.4 and -2.8 ± 0.6 respectively as shown by octanol extraction (Fig. S8).

**Figure S8**. LogD values for [^68^Ga]Ga-DFO and [^68^Ga]Ga-acetate detedmined by octanol extraction from phosphate buffered saline (PBS). Both [^68^Ga]Ga-DFO and [^68^Ga]Ga-acetate showed similar hydrophilic properties with logD_(octanol/PBS)_ values of -2.9 ± 0.4 and -2.8 ± 0.6 respectively.

**Serum stability study**

After protein precipitation with acetonitrile and centrifugation, <5% of the activity was in the precipitate, indicating that protein binding was minimal and that the supernatant samples used for iTLC and RP-HPLC analysis represent >95% of the total sample. [^68^Ga]Ga-acetate, by comparison, showed 70% of activity in the precipitate, indicating high protein binding (Figure S9A). iTLC of the supernatant was identical to that of control samples of [^68^Ga]Ga-DFO with >97% of activity eluting with Rf = 0.82 and 0.83 for the 5 and 60 minute incubation samples, respectively, consistent with radioactivity still being in the form of [^68^Ga]Ga-DFO. (Figure S9B

**Figure S9**: Preliminary serum stability measurements. **A**: percentage of activity in precipitate (protein binding) and supernatant after adding acetonitrile and centrifugation. **B** and **C**: iTLC of supernatant from protein precipitation and centrifugation after 5 and 60 min incubation with serum, respectively. In both samples the iTLC is indistinguishable from that of control samples of [^68^Ga]Ga-DFO incubated in PBS and contrasts with that of [^68^Ga]Ga-acetate incubated with serum which shows 100% of activity at Rf = 0 (panel **F**). **D** and **E**: radioHPLC of supernatant from protein precipitation and centrifugation after 5 and 60 min incubation with serum, respectively. In both samples the HPLC is indistinguishable from that of control samples of [^68^Ga]Ga-DFO incubated in PBS (panel **G**). iTLC mobile phase: 1M ammonium acetate in 1:1 v/v H_2_O and water (pH=7). HPLC mobile phase: water (A) and acetonitrile (B), each containing 0.1%TFA. HS, human serum.

and C). In contrast, when [^68^Ga]Ga-acetate was incubated with human serum the radioactivity remained at the origin (Figure S9F). RadioHPLC of the supernatant from the protein precipitation

experiment after [^68^Ga]Ga-DFO incubation with serum (Figure S9D and E) showed identical retention times to [^68^Ga]Ga-DFO incubated with PBS (Figure S9G), indicating that [^68^Ga]Ga-DFO remains chemically intact even after 60 minutes of incubation in serum.

**In vitro urine stability**

After incubation of [^68^Ga]Ga-DFO and [^68^Ga]Ga-acetate (control) with human urine for 30 and 60 min, respectively, radioHPLC showed that both are chemically altered. [^68^Ga]Ga-acetate showed at least 2 additional peaks eluting at ca. 4 and 7.5 min in addition to the peak at 2.3 min seen for untreated [^68^Ga]Ga-acetate (Fig. S10A). RadioHPLC of [^68^Ga]Ga-DFO after 60 min in urine showed, in addition to the expected peak at 10 min, a majority of activity in broad peaks between 2 and 9 min (Fig. S10B) indicating some instability and conversion to other radiochemical forms caused by components of urine.

**Figure S10**. RadioHPLC after incubation in human urine of [^68^Ga]Ga-acetate (**A**) and [^68^Ga]Ga-DFO (the latter compared with incubation in PBS) (**B**). Both show evidence of significant conversion to other chemical species. Mobile phase: water (solvent A) and acetonitrile (B) gradient with 0.1% TFA in both solvents A and B.

**Ex vivo mouse urine HPLC**

**Figure S11**. RadioHPLC analysis of mouse urine sampled ex vivo at 60 minutes post injection, showing no significant activity in the form of [^68^Ga]Ga-DFO. HPLC mobile phase: water (A) and acetonitrile (B), each containing 0.1%TFA.

**
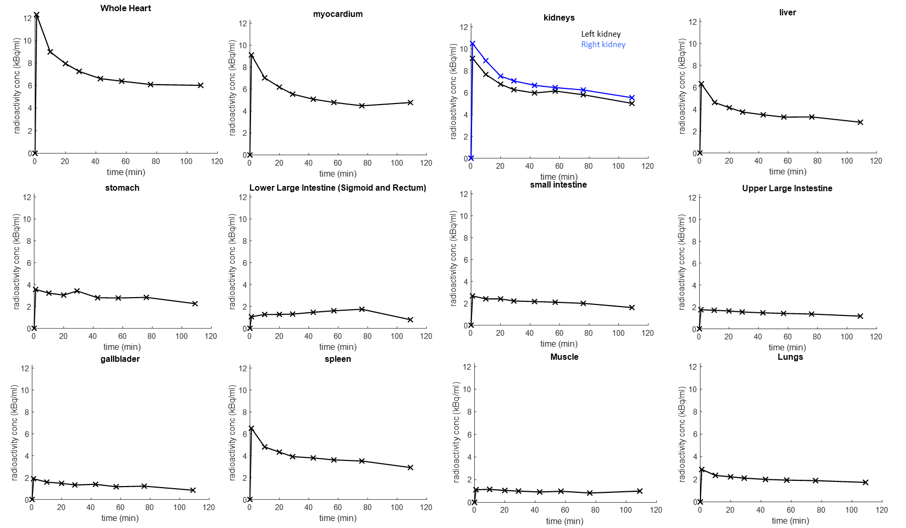
**

**Figure S12**. Time-activity curves (TACs) for all organs subjected to dynamic scanning in patient 2 (left to right, top row: whole heart, myocardium, kidneys, liver; middle row: stomach, lower large intestine, small intestine, upper large intestine; bottom row: gallbladder, spleen, muscle, lungs.

**Human dosimetry estimates extrapolated from mouse PET imaging**

**Table S1**: Organ doses calculated from the preclinical data extrapolated to the human adult phantom (73.7kg).

| **Organ** | **Calculated estimates (mSv/MBq)** |
| --- | --- |
| Adrenals | 6.29E-05 |
| Brain | 8.87E-06 |
| Breasts | 5.40E-04 |
| Gallblader wall | 0.00E+00 |
| LLI wall | 1.93E-03 |
| Small intestine | 7.17E-05 |
| Stomach wall | 1.54E-03 |
| UlI wall | 6.93E-05 |
| Heart wall | 0.00E+00 |
| Kidneys | 4.62E-05 |
| Liver | 2.03E-04 |
| Lungs | 1.40E-03 |
| Muscle | 6.26E-05 |
| Ovaries | 3.21E-03 |
| Pancreas | 6.53E-05 |
| Red Marrow | 1.23E-03 |
| Osteogenic Cells | 1.72E-04 |
| Skin | 1.08E-04 |
| Spleen | 6.29E-05 |
| Testis | 0.00E+00 |
| Thymus | 5.97E-05 |
| Thyroid | 6.05E-04 |
| Urinary Bladder wall | 1.36E-02 |
| Uterus | 9.86E-05 |
| **Effective Dose** | 2.51E-02 |

**Human PET studies**

**Table S2 Human absorbed radiation dose estimates based on human scans** Organ doses as calculated in OLINDA/EXM 1.1 from patient 2.

| **Organ** | **Calculated estimates (mSv/MBq)** |
| --- | --- |
| Adrenals | 5.26E-05 |
| Brain | 2.40E-05 |
| Breasts | 3.50E-04 |
| Gallblader wall | 0.00E+00 |
| LLI wall | 1.18E-03 |
| Small intestine | 5.01E-05 |
| Stomach wall | 1.49E-03 |
| UlI wall | 1.59E-04 |
| Heart wall | 0.00E+00 |
| Kidneys | 1.85E-04 |
| Liver | 1.16E-03 |
| Lungs | 4.01E-03 |
| Muscle | 4.46E-05 |
| Ovaries | 2.05E-03 |
| Pancreas | 4.99E-05 |
| Red Marrow | 7.65E-03 |
| Osteogenic Cells | 5.04E-04 |
| Skin | 5.99E-05 |
| Spleen | 1.12E-04 |
| Testis | 0.00E+00 |
| Thymus | 4.49E-05 |
| Thyroid | 3.62E-04 |
| Urinary Bladder wall | 4.51E-03 |
| Uterus | 5.38E-05 |
| **Effective Dose** | 2.41E-02 |

**Table S3. Biexponential fit of blood clearance parameters**

The table shows the parameters used to fit the following biexponential equation:

$$y=Ae^{-at}+Be^{-bt}$$

Where *y* = blood activity concentration (kBq/mL) at time *t*; *a*, *b* = fast and slow clearance rate constants, min^-1^; *t* = time, min

| **parameter** | **Patient 1** | **Patient 2** |
| --- | --- | --- |
| **A (fast coefficient)** | **15.32** | **16.97** |
| **a (fast rate, min^-1^)** | **0.123** | **0.090** |
| **Fast half-life (min)** | **7.7** | **5.1** |
| **B (slow coefficient)** | **9.70** | **12.85** |
| **B (slow rate, min^-1^)** | **0.002** | **0.002** |
| **Slow half-life (min)** | **348** | **320** |
