# Supplementary figures and images for: The 68Ga-siderophore approach to infection imaging: evaluation of [68Ga]Ga-DFO in patients with vascular graft infection
Source: Eur J Nucl Med Mol Imaging. 2026 Mar 6;53(6):4151–61. doi: 10.1007/s00259-026-07831-4 (PMC13121275; doi:10.1007/s00259-026-07831-4)

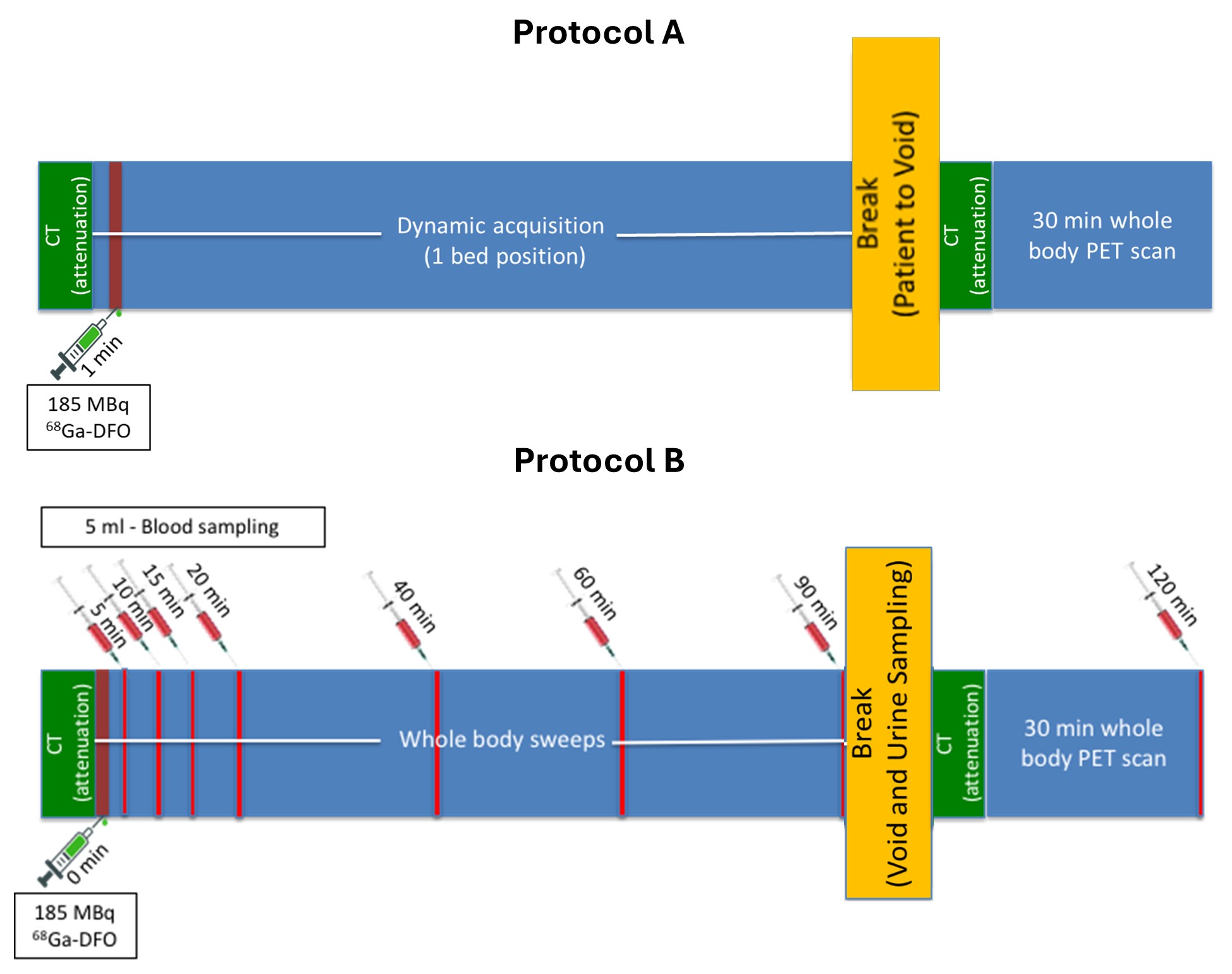

Supplement: Supplementary file 2 — Supplementary Material 2 [file 259_2026_7831_MOESM2_ESM.jpg]
